# Supplementary material for: MetaRibo-Seq measures translation in microbiomes
Source: Nat Commun. 2020 Jun 29;11:3268. doi: 10.1038/s41467-020-17081-z (PMC7324362; doi:10.1038/s41467-020-17081-z)
Supplement: Supplementary file 10 — Supplementary Data 7 [file 41467_2020_17081_MOESM10_ESM.zip › File2/Confidence_High_Taxonomy/87489_out.krona.html]

Javascript must be enabled to view this page.

members
magnitude
magnitudeUnassigned
count
unassigned
taxon
rank

87489\_out

1

SRS043701\_contig\_number\_contig-100\_3472.94062
5

3
2
superkingdom

1239
2
phylum

2
186801
class

order
186802
2

family
186804
1

1
1505652
genus

species

SRS049900\_contig\_number\_16717
36841
1

1
1950887

SRS015694\_contig\_number\_contig-100\_1.137914
species

1
32066
phylum

203490
1
class

1
203491
order

family
1129771
1

genus
34104
1


SRS018656\_contig\_number\_contig-100\_8214.46645
species
1
34105

superkingdom
1
2759

kingdom
1
4751

subkingdom
451864
1

1
4890
phylum

147538
1
subphylum

1
147550
class

subclass
222544
1

639021
1
order

1
81093
family

genus
1
228882


SRS044535\_contig\_number\_contig-100\_25226.69616
species
1
165778
